# Patient-reported outcomes of femoral osteotomy and total hip arthroplasty for osteonecrosis of the femoral head: a prospective case series study

**DOI:** 10.1186/s40064-016-3576-4

**Published:** 2016-10-26

**Authors:** Yusuke Kubo, Takuaki Yamamoto, Goro Motomura, Kazuyuki Karasuyama, Kazuhiko Sonoda, Yukihide Iwamoto

**Affiliations:** 1 Department of Orthopaedic Surgery, Graduate School of Medical Sciences, Kyushu University, 3-1-1 Maidashi, Higashi-ku, Fukuoka, 812-8582 Japan; 2 Department of Orthopaedic Surgery, Faculty of Medicine, Fukuoka University, 7-45-1 Nanakuma, Jonan-ku, Fukuoka, Japan; 3 Department of Orthopaedic Surgery, Kyushu Rosai Hospital, 1-1 Sonekita-machi, Kokuraminami-ku, Kitakyushu, 800-0296 Japan

**Keywords:** Patient-reported outcomes, Osteotomy, Total hip arthroplasty, Osteonecrosis of the femoral head, Oxford hip score, Short form 36

## Abstract

**Background:**

Patient-reported scoring systems have recently been used after surgical procedures. The purpose of this prospective study was to evaluate the patient-reported outcomes of femoral osteotomy and total hip arthroplasty (THA) for osteonecrosis of the femoral head (ONFH).

**Methods:**

Forty-two symptomatic ONFH patients with asymptomatic contralateral hip underwent either transtrochanteric anterior rotational osteotomy (ARO) or THA as a primary operation. Of these, 20 patients whose contralateral hips remained asymptomatic at the final follow-up (more than 1 year postoperatively) were recruited to participate in this study. Nine patients were treated with ARO (ARO group) and 11 patients were treated with THA (THA group). Both the Oxford hip score (OHS) and the short form 36 (SF-36) were evaluated preoperatively and at the final follow-up.

**Results:**

The preoperative OHS was 29.1 ± 10.9 and 21.9 ± 9.6 points in the ARO and THA groups, which significantly improved to 38.4 ± 9.4 and 40.3 ± 5.1 points at the final follow-up, respectively. The preoperative physical component summary score was 30.8 ± 12.8 and 17.8 ± 14.5 points in the ARO group and THA groups, which significantly improved to 44.5 ± 10.6 and 43.3 ± 10.4 points at the final follow-up, respectively. The preoperative mental component summary score was 48.0 ± 8.5 and 48.6 ± 11.3 points in the ARO and THA groups, both of which remained unchanged at the final follow-up.

**Conclusions:**

The short-term patient-reported outcomes of this study suggested that both ARO and THA for ONFH resulted in significantly improved postoperative hip joint function.

## Background

Osteonecrosis of the femoral head (ONFH) often leads to collapse of the femoral head, which usually causes severe hip pain and thus requires some kind of surgical treatment (Mankin 1992; Mont et al. 1995; Fukushima et al. 2010). Surgeries for collapsed ONFH are classified in two major categories: total hip arthroplasty (THA) and joint-preserving surgeries (Sugioka 1978; Mont et al. 1995). The worldwide standard surgical procedure for ONFH is THA. This procedure has a wide application for collapsed ONFH, especially in cases at advanced stages of ONFH (Wiklund and Romanus 1991; Mont et al. 1995). On the other hand, in the early postcollapse stages of ONFH, hip joint preservation remains a preferred treatment option.

Transtrochanteric anterior rotational osteotomy (ARO), developed by Sugioka in 1972, is one of the joint-preserving alternatives for ONFH (Sugioka 1978). In patients with necrotic lesions of the anterosuperior aspect of the femoral head, the anterior rotation of the femoral head enables relocation of the weight-bearing region of the hip joint to the intact posterior aspect of femoral head (Sugioka 1978). To date, many studies have focused on ARO with the objective of improving the performance of surgery, which translates into an improvement of the joint preservation rate (Inao et al. 1999; Mont et al. 2006). In these studies, physician-reported scoring systems were commonly used as the surgical assessment.

To the best of our knowledge, no previously published studies have prospectively examined the perioperative patient-reported outcomes of symptomatic ONFH in patients with an asymptomatic contralateral hip who underwent either ARO or THA as the primary operation. These evaluations are considered to be useful for patients at the early postcollapse stages, particular in the decision-making process of whether to preserve the joint or not. In this study, we prospectively evaluated the patient-reported outcomes of ARO and THA for ONFH.

## Methods

### Patients

Forty-two symptomatic ONFH patients with asymptomatic contralateral hip were prospectively assessed for perioperative patient-reported outcomes. They underwent either ARO or THA as the primary operation for ONFH at our institution between June 2009 and June 2014. Of these, 20 patients whose contralateral hip remained asymptomatic at the final follow-up (which was variable, but lasted more than 1 year postoperatively) were recruited to participate in this study. They consisted of nine patients treated with ARO (ARO group) and 11 patients treated with THA (THA group) (Table 1). In the ARO group, there were eight men and one woman with a mean age of 38 years (range: 28–45) at the time of surgery and a mean body mass index (BMI) of 24.1 kg/m^2^. In the THA group, there two men and nine women with a mean age of 45 years (range: 29–60) at the time of surgery and a mean BMI of 21.8 kg/m^2^. The mean preoperative period, defined as the time from the onset of the hip pain until surgery, was 4.8 and 9.3 months in the ARO and THA groups, respectively. The mean postoperative follow-up period was 2.5 years (range: 1–6) and 1.7 years (range: 1–4) in the ARO and THA groups, respectively.

**Table 1:** Demographic characteristics of the two groups

Patient no.	Group	Age (years)	Sex	Etiology
1	ARO	38	Male	Alcohol
2	ARO	41	Male	Alcohol
3	ARO	45	Male	Alcohol
4	ARO	36	Male	Alcohol
5	ARO	28	Male	Steroids
6	ARO	38	Male	Idiopathic
7	ARO	32	Male	Steroids
8	ARO	40	Male	Steroids
9	ARO	40	Female	eroids
10	THA	29	Female	Steroids
11	THA	57	Female	Steroids
12	THA	60	Male	Alcohol
13	THA	32	Female	Steroids
14	THA	47	Female	Steroids
15	THA	55	Male	Alcohol
16	THA	31	Female	Idiopathic
17	THA	50	Female	Alcohol
18	THA	30	Female	Steroids
19	THA	58	Female	Steroids
20	THA	41	Female	Alcohol

*ARO* transtrochanteric anterior rotational osteotomy, *THA* total hip arthroplasty

The diagnosis of ONFH was based on the diagnostic criteria, including findings on plain radiograph, magnetic resonance imaging, and bone scans (Sugano et al. 1999). According to the classification system of the Japanese Investigation Committee of Health and Welfare, five hips in the ARO group and six in the THA group were classified as stage 3A, meaning a collapse of less than 3 mm. Four hips in the ARO group and four hips in the THA group were stage 3B, indicating a collapse of 3 mm or more. One hip in the THA group was stage 4, indicating the presence of osteoarthritic change (Sugano et al. 2002). According to the location of the necrotic lesion, five hips in the ARO group and three hips in the THA group were classified as type C1, indicating that the necrotic lesion occupied more than two-thirds of the weight-bearing portion, but did not extend to the acetabular edge. Four hips in the ARO group and eight hips in the THA group were classified as type C2, indicating that the necrotic lesion extended to the acetabular edge (Sugano et al. 2002).

### Indications for surgeries

**Fig. 1:**
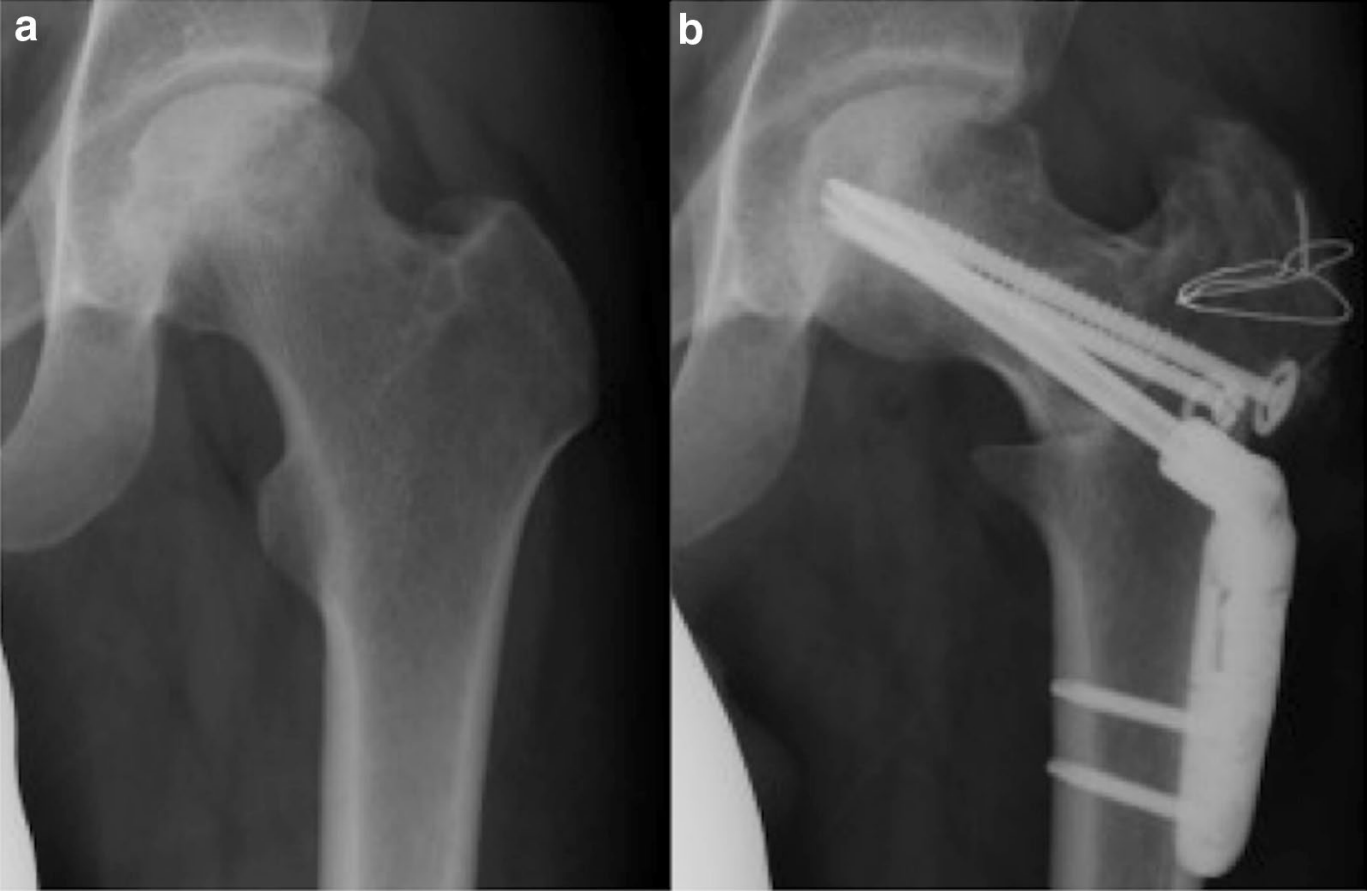
A 28-year-old male with unilateral ONFH treated with ARO (Patient 5). **a** Preoperative anteroposterior radiograph shows left femoral head collapse and bone sclerosis, which indicates ONFH with stage 3A and type C1. **b** Anteroposterior radiograph after ARO of the left hip

**Fig. 2:**
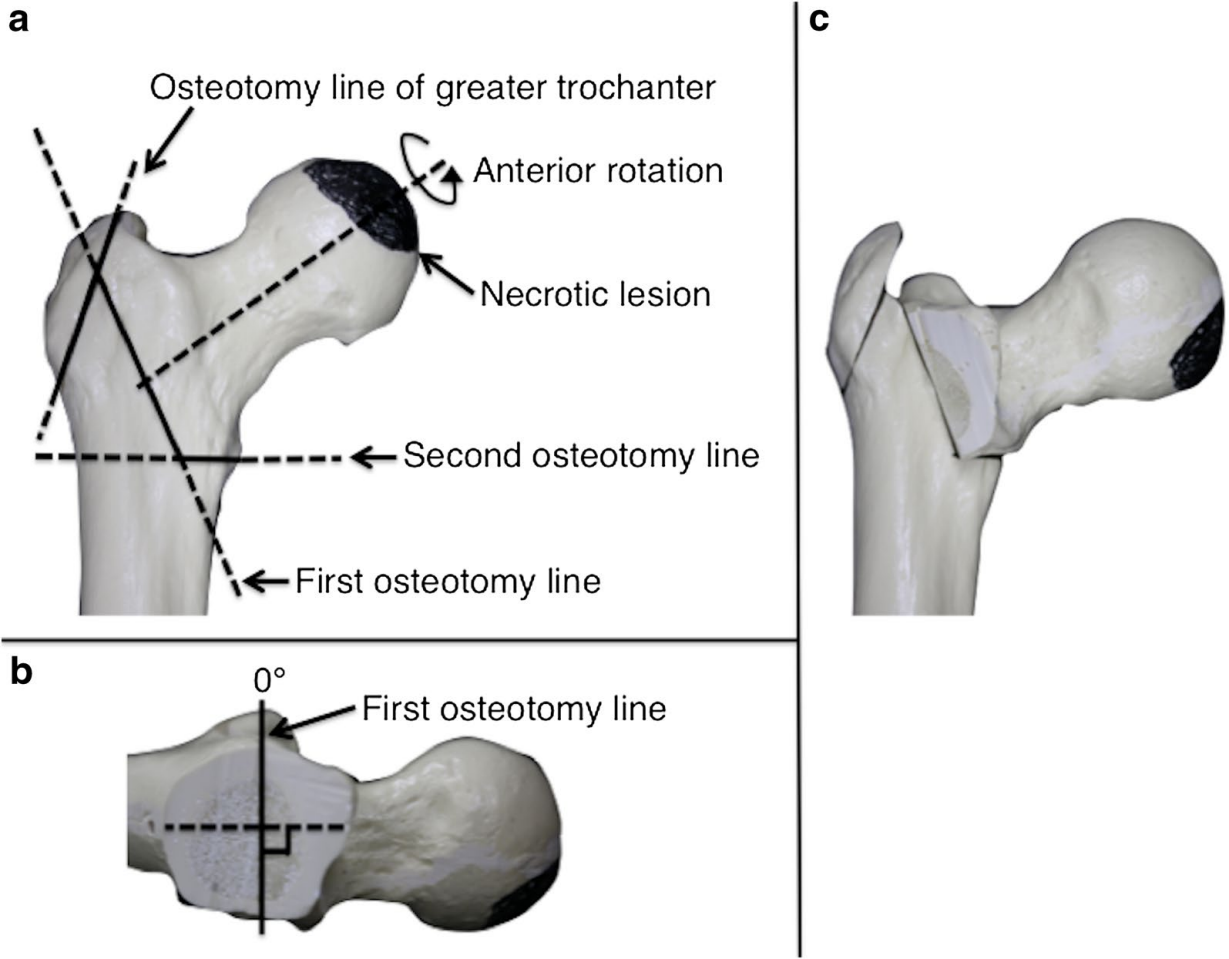
Three-dimensional illustrations of the technique for ARO using a bone model. Anteroposterior **a** and lateral **b** views show the two osteotomy lines. The first osteotomy is made in the plane perpendicular to the femoral neck, and the second line is made directing to the first osteotomy plane, just above the lesser trochanter. Anteroposterior view **c** showing the bone model after 90° of anterior rotation

In general, the indications for ARO were less than 60 years of age and stage 3A or 3B ONFH with one-third or more of intact area in the posterior region of the femoral head. ARO was performed for male and female patients who preferred hip joint preservation after both ARO and THA were explained to them. Twelve of 20 patients had indications for ARO in this study. Of these patients, ARO was performed for nine patients. The surgical procedure of ARO was performed according to the originally described method (Sugioka 1978). Two Kirschner wires were inserted into the femoral neck through the center of the femur, perpendicular to the plane of the femoral neck. After radiographic control, the first osteotomy was made in the plane of the Kirchner wires. The second osteotomy was made directing to the first osteotomy plane, just above the lesser trochanter. The proximal fragment was then rotated such that the new weight-bearing areas are well apposed. Two or three lag screws were then inserted to maintain fixation in this position (Figs. 1; 2). Patients usually began using wheelchairs 2 days after surgery. Partial weight-bearing walking was permitted approximately 5 weeks after surgery, followed by gradual increase in weight bearing. Full weight-bearing walking was basically permitted at around 6 months postoperatively. On the other hand, THA is indicated for patients who have no indication or do not wish to undergo ARO. Full weight-bearing walking was usually permitted a few days after surgery.

### Clinical assessment

The preoperative assessment was performed at the time of hospital admission for surgery, and the postoperative assessment was performed at the time of final follow-up in the outpatient clinic.

The Japanese Orthopaedic Association (JOA) hip score evaluates pain (0–40), gait (0–20), activities of daily living (0–20), and range of motion (0–20). The possible range of values is 0–100, with the higher values reflecting a better state (Takeda et al. 2006).

The Oxford hip score (OHS) is a disease-specific 12-item questionnaire evaluating both pain (6 items) and physical function (6 items) of the hip joint in relation to daily activities (Dawson et al. 1996). The six items (OHS 1, 6, 8, 10, 11, and 12) indicate pain severity, while the other six items (OHS 2, 3, 4, 5, 7, and 9) indicate physical function. The two items, OHS 5 and 7, are related to the bilateral hip function. This questionnaire was originally written in English, and Uesugi et al. translated the questionnaire into Japanese (Uesugi et al. 2009). Their translation was subsequently validated by a back translation as described in a previous report (Uesugi et al. 2009). The original version from 1996 was updated in 2007 introducing a new scoring system. The new scoring was supported by the original authors: item scores range from 0 to 4 (worst to best) with overall scores ranging from 0 to 48, where a score of 48 is the best possible score (Nilsdotter and Bremander 2011). We also modified the classification of OHS described by Kalairajah et al. as follows: excellent, 42–48 points; good, 34–41 points; fair, 27–33 points; and poor, less than 27 points (Kalairajah et al. 2005).

The Medical Outcomes Study Short Form 36 (SF-36; SF-36v2 Standard, Japanese) was used to evaluate quality of life. It is composed of 36 items that measure general health on eight subscales (Fukuhara et al. 1998). We derived a physical component summary (PCS) score and a mental component summary (MCS) score from the SF-36; then, an adjusted score was calculated by norm-based scoring, which facilitates comparison of each score with that of the Japanese population norm.

### Statistical analysis

The JOA score, OHS, and SF-36 were compared using Student’s *t* test. The distribution of ONFH etiology in the two groups was compared using the Chi square test. P values less than 0.05 were considered significant. All statistical analyses were performed using the JMP Pro 11 software package (SAS Institute, Cary NC, USA).

## Results

Regarding the etiology of ONFH, corticosteroid use was associated with four hips in the ARO group and six hips in the THA group. Alcohol abuse was associated with four hips in each group. One hip in each group did not have any associated factors (P = 0.90) (=idiopathic ONFH).

**Fig. 3:**
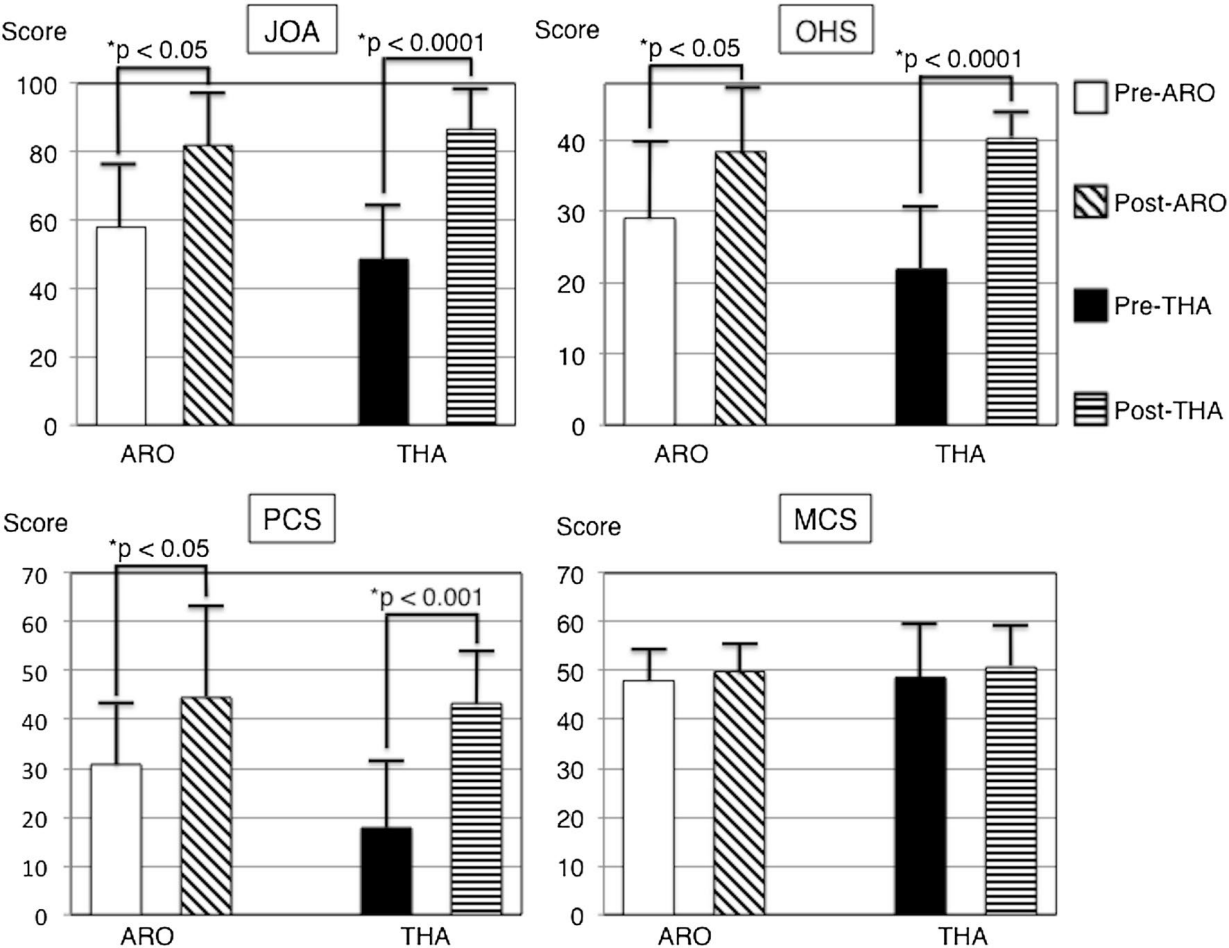
Comparison of JOA score, OHS, PCS and MCS scores in the ARO and the THA groups. *JOA* Japanese Orthopaedic Association, *OHS* Oxford hip score, *PCS* physical component summary, *MCS* mental component summary, *ARO* transtrochanteric anterior rotational osteotomy, *THA* total hip arthroplasty

The preoperative JOA score was 58.1 ± 20.5 and 48.6 ± 16.7 points in the ARO and THA groups, which significantly improved to 81.9 ± 14.5 and 86.6 ± 10.6 points at the final follow-up, respectively (Fig. 3). During the follow-up period, one patient in the ARO group presented separation of the greater trochanter 1 week postoperatively, resulting in osteosynthesis 4 weeks postoperatively. There were no other complications in both groups.

**Fig. 4:**
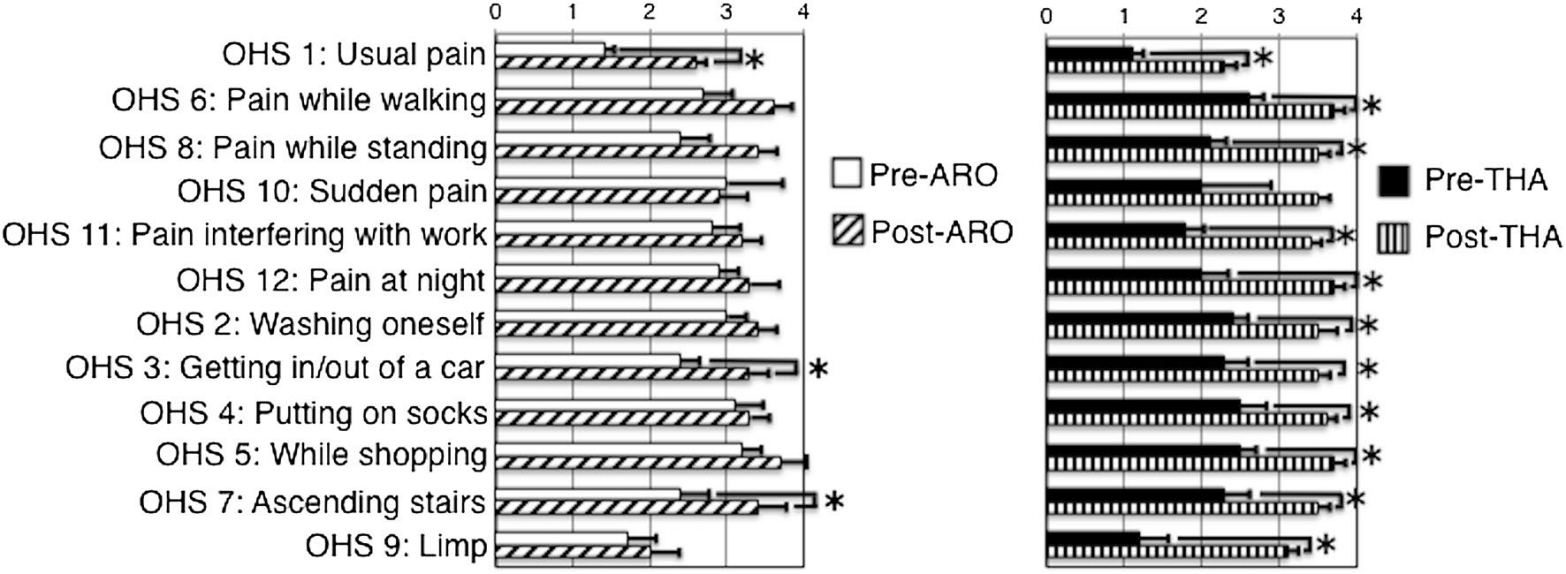
Comparison of perioperative OHS subscales in the ARO and the THA groups. *P value < 0.05. *Error bars* standard deviation. *OHS* Oxford hip score, *ARO* transtrochanteric anterior rotational osteotomy, *THA* total hip arthroplasty

The preoperative OHS was 29.1 ± 10.9 points (excellent: 1 hip, good: 1 hip, fair: 4 hips, and poor: 3 hips) in the ARO group and 21.9 ± 9.6 points in the THA group (good: 3 hips, poor: 8 hips), which significantly improved to 38.4 ± 9.4 points (excellent: 5 hips, good: 3 hips, and poor: 1 hip) and 40.3 ± 5.1 points (excellent: 5 hips, good: 4 hips, and fair: 2 hips) at the final follow-up, respectively (Fig. 3). At the final follow-up, there were significant improvements in ‘usual pain’, ‘getting in/out of a car’, and ‘ascending stairs’ in the ARO group. Other items were also improved although not significantly. Notably, only the limp item was rated with a low score in this group. In the THA group, significant improvements were found in all of the items, except for ‘sudden pain. The ‘sudden pain’ item was also improved although not significantly (Fig. 4).

**Fig. 5:**
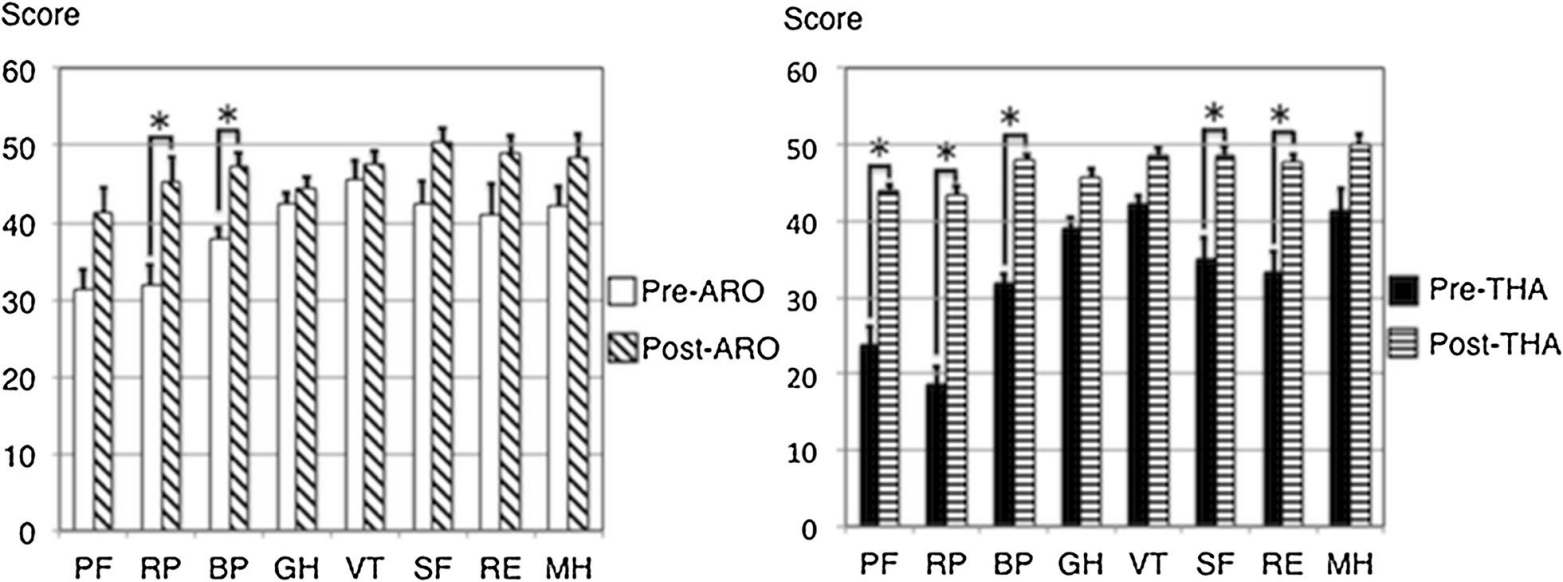
Comparison of perioperative SF-36 subscales in the ARO and the THA groups. *P value < 0.05. *Error bars* standard deviation. *SF-36* medical outcomes study short form 36; *ARO* transtrochanteric anterior rotational osteotomy, *THA* total hip arthroplasty

The preoperative PCS score was 30.8 ± 12.8 and 17.8 ± 14.5 points in the ARO and THA groups, which significantly improved to 44.5 ± 10.6 and 43.3 ± 10.4 points at the final follow-up, respectively. On the other hand, the preoperative MCS score was 48.0 ± 8.5 points in the ARO group and 48.6 ± 11.3 points in the THA group, both of which remained unchanged at the final follow-up (Fig. 3). Regarding each SF-36 subscale, there were significant improvements in two of the eight subscales (‘role physical’ and ‘bodily pain’) in the ARO group, while in the THA group, five of the eight subscales (‘physical functioning’ ‘role physical’ ‘bodily pain’ ‘social functioning’, and ‘role emotional’) were found to be significantly improved (Fig. 5). In comparison with the PCS score for the normal Japanese population, four hips (44.4%) in the ARO group and four hips (36.4%) in the THA group were within the Japanese norm (PCS score of 50). Regarding the MCS score for normal Japanese population, three hips (33.3%) in the ARO group and five hips (45.5%) in the THA group were within the Japanese norm (MCS score of 50).

## Discussion

Reportedly, patients that undergo THA are generally satisfied with the outcomes (Wiklund and Romanus 1991; Learmonth et al. 2007), while the satisfaction level of patients that undergo ARO is relatively unknown. In the current prospective study, we found significant improvements in the hip joint functions according to patient-reported outcomes not only in patients who underwent THA but also in those who underwent ARO during a short-term follow-up period. Although full weight-bearing walking is basically permitted at around 6 months after ARO, the results of the current study show that joint-preserving surgery needs to be considered for young patients in order to avoid or delay THA (Ikemura et al. 2009).

Several studies have reported patient-reported outcomes of ONFH treatment (Seki et al. 2008; Motomura et al. 2010; Kang et al. 2013). Seki et al. evaluated the SF-36 in 41 ONFH patients at a mean follow-up of 5.2 years after transtrochanteric rotational osteotomy (TRO) and in 19 patients at a mean follow-up of 4.1 years after THA. They reported that average PCS and MCS scores after TRO were 39.4 and 49.6 points, and average PCS and MCS scores after THA were 39.1 and 50.3 points, respectively (Seki et al. 2008). Kang et al. evaluated the SF-36 in 24 systemic lupus erythematosus patients with ONFH at a mean follow-up of 67.5 months after THA, reporting average PCS and MCS scores after THA of 42.2 and 49.5, respectively (Kang et al. 2013). In our study, the postoperative PCS score was 44.5 in the ARO group and 43.3 in the THA group, both of which were slightly higher than those in the previous reports. These slight differences in outcomes may be explained by the fact that participants of the current study had symptomatic ONFH with asymptomatic contralateral hip. In our study, 22 (52.4%) of 42 patients presented contralateral hip pain during the follow-up period, and thus were excluded from the present analysis. However, in the previous reports, ONFH patients with symptomatic contralateral hip were not strictly excluded, which might lead to lower PCS scores.

In the current study, of all OHS items assessed by patients in the ARO group at the final follow-up, the only item that did not reflect improvement was the ‘limp’ item. This finding indicates that limping was often experienced by patients that underwent ARO. In ARO surgery, a varus positioning of the femoral neck-shaft angle is frequently intended to acquire enough intact weight-bearing area of the femoral head, which can thereby cause shortening of the leg (Sugioka 1978; Dean and Cabanela 1993; Ha et al. 2010). We therefore consider that one of the major reasons for limping after ARO is the resulting leg length discrepancy caused by the intended varus neck-shaft-angle created during ARO. For this reason, surgeons should clearly explain to their patients that limping may present secondary to a leg length discrepancy after ARO.

The main limitation of this study was the small sample size. However, we only included ONFH patients with asymptomatic contralateral hip during the follow-up. We therefore believe that the current results reflect the real outcomes of the surgery performed without any influence of the contralateral hip symptoms. A future multicenter study should be conducted with a similar protocol to validate our results. Second, the postoperative follow-up period was short in this study (range: 1–6 years). However, the postoperative complication in both groups was only one instance of greater trochanter separation. Therefore, we believe that these differences have little effect on the result of this study. Third, we evaluated the functional status only at the time of hospital admission for surgery and final follow-up. Considering that full weight-bearing walking was basically permitted at around 6 months after ARO, at least 1 year seems necessary to evaluate postoperative functional outcomes.

## Conclusions

The short-term patient-reported outcomes of this study suggested that both ARO and THA for ONFH resulted in significantly improved postoperative hip joint function.

## Abbreviations

THA: total hip arthroplasty
ONFH: osteonecrosis of the femoral head
ARO: transtrochanteric anterior rotational osteotomy
OHS: Oxford hip score
SF-36: short form 36
BMI: body mass index
JOA: Japanese Orthopaedic Association
PCS: physical component summary
MCS: mental component summary
TRO: transtrochanteric rotational osteotomy
